# The hidden burden of medical testing: public views and experiences of COVID-19 testing as a social and ethical process

**DOI:** 10.1186/s12889-022-14217-2

**Published:** 2022-09-30

**Authors:** Alice Street, Shona J. Lee, Imogen Bevan

**Affiliations:** University of Edinburgh, Edinburgh, Scotland

**Keywords:** COVID-19, Testing, Values, Ethical dilemmas, Scotland, Trust

## Abstract

**Background:**

In May 2020, the Scottish Government launched Test and Protect, a test, trace and isolate programme for COVID-19 that includes a PCR testing component. The programme’s success depended on the willingness of members of the public to seek out testing when they experienced symptoms and to comply with guidelines on isolation should they test positive. Drawing on qualitative interview-based research, this paper analyses public understandings, expectations, and experiences of COVID-19 testing during the early stages of the programme. ﻿Through anthropological and sociological analysis of the findings we aim to contribute to social understandings of COVID-19 testing practices; and to inform the design of population level testing programmes for future pandemics.

**Methods:**

Between 7 July and 24 September 2020, 70 semi-structured interviews were conducted with members of the general public (aged 19–85) living in the Lothian region of Scotland. Interviews were held online or by telephone, were transcribed verbatim and analysed using thematic analysis informed by anthropological and sociological theories of medical testing.

**Findings:**

Social relationships and ethical considerations shape testing practices at every stage of the testing process. Members of the public viewed testing as a civic duty to society and moral duty to friends, family, and colleagues. However, the testing process also placed a significant social, economic, and practical burden on the individual and sometimes generated competing obligations. Many participants experienced a disconnect between the government’s portrayal of testing as easy and the everyday burden of testing.

**Conclusions:**

COVID-19 testing is experienced as a social process shaped by multiple relationships and ethical considerations. The full burden of testing should be considered in the design of future testing programmes.

**Supplementary Information:**

The online version contains supplementary material available at 10.1186/s12889-022-14217-2.

## Background

On 28 May 2020, the Scottish Government launched Test and Protect, the national contact tracing and testing service for COVID-19. At the time, no approved treatments or vaccines for the disease were available and diagnostic testing, coupled with isolation guidelines and other Non-Pharmaceutical Interventions (NPIs), were the best available tools to reduce community transmission [1–3]. One component of the Test and Protect programme was a polymerase chain reaction (PCR) based community testing programme, provided through a UK-wide network of testing centres and laboratories. Everyone experiencing COVID-19 symptoms (aged five and over) was asked to book a test through the online booking system, attend a dedicated testing centre, and isolate along with members of their household while they awaited their test result. Those who tested positive, their household members, and any close contacts were instructed to isolate for 14 days.

The willingness of members of the public to voluntarily seek testing was essential to the success of Test and Protect. Yet the expectations that testing guidance placed on people were unprecedented: individuals were asked to continually be alert for symptoms of COVID-19; apply changing testing criteria to those symptoms; book a test; organise transport and other logistics for attending a testing centre; collect their own (or their child’s) sample using a nasopharyngeal swab; isolate for 24 h or more while awaiting a result; and, in the event of a positive result, isolate for 14 days from the date of onset of symptoms. Furthermore, testing for COVID-19 was not a one-off event, as individuals were expected to maintain their vigilance of COVID-19 symptoms and potentially get tested on multiple occasions. Despite these expectations, community-based testing for COVID-19 has no direct medical benefits for the individuals involved. Understanding how people balance the public benefits of COVID-19 testing with the personal costs involved is therefore essential to understanding facilitators and barriers to effective implementation of COVID-19 testing and other population level testing programmes in the future.

A substantial body of work published over the course of the pandemic has revealed the importance of social and economic factors in testing uptake and adherence to public health guidelines. A scoping review of research on knowledge, attitudes, and behaviours related to COVID-19 testing, published in late 2021, summarised key findings from this research [4]. The review found that members of the public in multiple national settings were widely accepting of COVID-19 testing technologies and were willing to undergo testing. Social solidarity and a sense of civic responsibility were identified as important motivators to seek testing. Multiple studies also revealed barriers to testing uptake, including logistical issues [5–9], questions of accessibility [5, 6], the physical discomfort associated with collecting a sample [10–13], ﻿economic pressures and anxieties [6, 10, 14, 15], and symptom identification [5, 6, 15–20]. ﻿ ﻿Lack of trust in government bodies to deliver and manage testing was identified as a key barrier to testing in several studies [18, 21], a finding that has been repeated in recent research on the role of governmental and interpersonal trust in vaccine uptake [22].

The scoping review also found that, apart from a few exceptions [23–29], social studies of COVID-19 testing have so far been dominated by quantitative research methods, especially the use of cross-sectional surveys. ﻿Quantitative studies can provide crucial insight into how widespread a particular belief or behaviour might be, and can identify significant associations between self-reported beliefs/behaviours and other factors, such as demographic characteristics. However, the multiple-choice format of many surveys can prevent further probing of respondents’ experiences and thus inhibit access to the meanings and personal significance of participants’ statements and choices within their everyday lives and relationships [30].

In-depth, qualitative research on testing by anthropologists and sociologists has shown that testing is often experienced as a process that is extended in time and space, rather than a one-off event [31–33], and that personal testing decisions are embedded in people’s everyday lives, value systems and social relationships [34–38]. In particular, testing decisions often generate moral uncertainties and place new burdens of responsibility on individuals, for example to undergo testing, to weigh up and mitigate the impact of test results on others, and to disclose test results to the state and/or family, friends and colleagues [36–42]. Understanding the social and ethical dimensions of testing is especially important for voluntary population-level testing programmes, like that for COVID-19, which offer little medical benefit to the individual and yet entail significant social and economic costs. In this qualitative interview-based study, we therefore focus on the ethical and social considerations that PCR testing for COVID-19 posed for people in Scotland during the early stages of the pandemic, and explore how they sought to resolve those dilemmas in their testing decisions and practices.

Drawing on theoretical approaches to ethics and morality in anthropology [43–48] we understand ethics as people’s everyday moral striving, self-reflection and practical judgement in relation to moral norms, social codes, and notions of what constitutes the ‘right’ behaviour. We draw in particular on theoretical understandings of ethical dilemmas as instances of ‘moral breakdown’ that represent heightened moments for understanding people’s value systems, moral reasoning and contextual decision making [49]. An anthropology of ethics approach to testing therefore highlights the moral and social consequences for participants of choosing to test, as well the ethical dimensions of waiting for test results, and in trying to interpret and act on results in the best possible way. Bringing together this approach to testing as a form of ethical practice with recent anthropological research on health-seeking as a form of work [50] and the intensification of informal and voluntary health-related work in public health emergencies [51], we reflect on the extent to which ethical dilemmas and decision making can be considered a hidden burden of voluntary testing programmes.

We suggest that the insights that qualitative, anthropological and sociological research can provide to subjective experiences of testing have a considerable but, until now, under-utilised contribution to make to the design of appropriate COVID-19 testing interventions. For example, it can show that members of the public who could be simply glossed as ‘non-compliant’ are likely to be striving for other kinds of ‘good’ in the midst of multiple and often conflicting responsibilities and obligations [43, 44]. The aim of this study is therefore to contribute to social understandings of COVID-19 testing so as to inform testing policy for COVID-19 and for future pandemics.

## Methods

### Study design and participants

In this study we undertook in-depth, semi-structured interviews with members of the public living in the Lothian region of Scotland to explore how they understood, viewed, and experienced the Test and Protect programme during its early months of operation. Lothian was selected for its large population size (approximately 900,000), and diversity (for example 17.9% of the population of Edinburgh, the region’s largest city, identify as belonging to an ethnic minority according to the Scottish Census). Lothian also benefited from an established network of university and NHS laboratories, enabling it to develop a regional testing infrastructure early on in the pandemic [52]. The region had high rates of COVID-19 testing participation [53], and high rates of positive cases [54], and therefore offered a large pool of potential participants with testing experiences at the time of the study. Furthermore, at the time of the study, travel restrictions were in place and therefore recruitment activities such as distributing posters and information leaflets were limited to a defined local area within which study staff resided at the time.

Inclusion in the study was open to all adult members of the general public — not only those who had participated in testing — to ensure we accessed a wide range of views, including those of people who had not yet accessed PCR testing and those who were reluctant to get tested. A semi-structured interview topic guide was informed by: a review of anthropology and sociology literature on testing [42]; background interviews with nine key stakeholders in Scotland’s COVID-19 testing response (research scientists, laboratory managers, and health board management staff); and a background overview of COVID-19 testing policies and services in the UK and Scotland. The topic guide covered personal experiences of COVID-19, understandings and expectations of tests, testing experiences and meanings, test results and behaviour, and opinions about the UK and Scottish government testing strategies. For participants who had direct experience of testing (defined as having sought a COVID-19 test, or having had a close family member or household member seek a test), a sub-set of questions were asked to prompt people’s full testing ‘story’ [55, 56]. The topic guide was piloted with eight members of the public prior to finalisation by the team. To meet the requirements of a rapid research response, pilot participants were selected using convenience sampling and included six women and two men who were employed in the public, private and third sector. All the pilot participants identified as white, a limitation that resulted from the rapid response methodology of the study. No patient and public involvement activities took place in this study. A molecular diagnostics and molecular epidemiology expert advised on study design and protocols. As residents of Lothian region, IB and AS were patient participants in the Test and Protect programme at the time of the research. Preliminary findings from the study were published and made available for comment on the project website and presented at a public webinar.

All eligible participants who completed a registration questionnaire prior to 24 September 2020 were invited for interview. The data collection end date was determined by data saturation, defined by the point at which no new information or themes were emerging from the data.

Ethics approval was granted by the University of Edinburgh College of Humanities and Social Science Research Governance Committee (CAHSS200605). Informed consent was obtained from all participants in the study. Participants did not receive financial compensation.

### Procedures

Recruitment and interviews took place remotely. Participants were recruited through social media channels Twitter and Instagram via the handle @testing_trust, University of Edinburgh mailing lists, Facebook community groups, and study posters displayed on public noticeboards in Edinburgh (in supermarkets, churches, community centres), which provided a link to the project blog where participants could access information about the study and register to participate. Several community-based charities and organisations working directly with minority groups in the Lothian region assisted with dissemination of information about the project to their members.

Volunteers were given access to a participant information sheet explaining the aims, methods and procedures of the study and filled in a short registration form on our website, which included demographic data, postcode data, contact details, and written consent to participate, before choosing a slot for an interview to take place either online via Microsoft Teams or by telephone. Participants were eligible to be interviewed if they were aged over 18, lived in Lothian, and had provided consent and contact details.

Participants who agreed to be interviewed were re-briefed at the beginning of the interview on the information provided in the participant information sheet, and reassured that they could pause or end the interview at any time. Verbal consent was obtained in addition to the written consent given at the time of submitting their form. Hand-written notes were kept by both interviewers (SJL and IB) to capture immediate observations, the interview context and ideas about themes. Audio recordings were transcribed by a professional transcriber and checked for errors by the interviewers before being imported into NVivo.

All researchers have training in qualitative methods and several years of experience in interview-based research. At the time of the study, SJL and AS held PhDs and IB held an MSc Research in Medical Anthropology and four years training in anthropological research at a PhD level. AS was employed as a Senior Lecturer in Anthropology at the University of Edinburgh, SJL was employed as a Research Fellow at the University of Edinburgh. IB was employed as a Research Assistant at the University of Edinburgh. All researchers identify as female.

### Qualitative analysis

We followed Braun and Clarke’s six phase approach to thematic analysis: familiarisation, generating initial codes, searching for themes, reviewing themes, identifying and naming themes, and reporting [57]. This approach was informed by inductive narrative analysis approaches in medical anthropology and sociology that examine interviews as ‘stories of experiences’, which involve self-generated meanings, rather than as sources of information [56, 58, 59], and focus on the social positioning and the moral uncertainties generated by health-care encounters [55, 59, 60]. Early transcripts underwent a round of close reading and re-reading by both SJL and IB to familiarise themselves with the data, before conducting preliminary coding of the first 10 transcripts and co-developing a preliminary analysis document where initial code categories and labels were documented and reviewed. The code categories were discussed by all team members and codes were either revised or removed to reduce overlap, minimise bias and ensure interpretation and labelling was consistent. All transcripts were then closely read and coded by SJL using the qualitative analysis software package NVivo (version 12) and then reviewed by the full team in weekly meetings, with the final coding framework and themes defined and agreed by consensus. A sample of transcripts were read by IB and AS prior to the meetings to check for biases or omissions in the coding. A thematic table (tables S1, S2, S3, S4, S5, and S6, supplementary material) was then created by the team to organise themes and interpretive codes alongside illustrative quotes.

## Results

One hundred nine people completed the short registration survey by 24 September 2020, 105 of whom were eligible to participate in the study. The majority of people who completed the form and were eligible heard about the study through social media (75), followed by word of mouth (20), other (9) and local news (1). All eligible participants were invited for interview, and 70 participants aged 19–85 were successfully contacted and interviewed. 27 participants had a direct experience of testing. Two participants had received a positive test result. Interviews were conducted both over the telephone (46) and online (24), and lasted from 17 to 120 min (mean 57 min). Only the interviewer and participant were present at the interview. Demographic characteristics of the study participants are provided in Table 1. The age and gender characteristics of the sample reflect demographic trends in testing data at the time the study commenced, which showed the highest levels of testing uptake and positive cases to be among women aged between 30–65 [61]. We identified six thematic areas related to different stages in the testing process: perceived benefits of testing; expectations of testing provision and providers; interpretation of symptoms and decisions to test; experiences and perceptions of accessing testing; sample collection; and experiences of receiving, interpreting, and acting on test results. Within each thematic area we identified several sub-themes and associated social and ethical dimensions that interviewees perceived as important (Table 2). A more detailed table of themes and illustrative quotes for each theme can be viewed in the Supplementary Materials (Tables S1, S2, S3, S4, S5, and S6).

**Table 1:** Characteristics of study participants (*n* = 70)

		**Numbers of participants**
**Gender**	Male	21
	Female	49
**Age**	18–30	10
	30–44	27
	45–65	25
	> 65	8
**Ethnicity**	White Scottish, White British, White Irish, White Other	62
	Mixed or Multiple Ethnic Groups Asian, Asian Scottish or Asian British	3 5
**Interview period**	July 2020	18
	Aug 2020	24
	Sept 2020	28
**Experience of testing**	Experience of testing by the interviewee and/or a family member	27
	No direct experience	43

**Table 2:** Thematic summary of findings

Theme	Sub-theme	Social and ethical considerations
**Perceived benefits of testing**	Creates knowledge of personal COVID-19 status	• Responsibility for one’s own health and wellbeing • Responsibility to keep people you know safe • Responsibility to the wider community to reduce transmission/contribute to a collective response. insert bullet point Social contract with the state • Fear of moral judgment by others
	Keeps friends, family, and colleagues safe	
	Avoids social stigma	
	Reduces levels of transmission in population	
	Contributes to disease surveillance and policy/planning	
**Expectations of, and trust in, testing provision and providers**	Expectations about the availability of testing	• Obligation of government to make testing available and accessible • Obligation of citizens to access testing when symptomatic • Responsibility of citizens not to waste public resources • Making profit from a public good considered unethical
	Concerns about wasting tests	
	Concerns about testing, or testing data, as a commodity rather than a public good	
**Experiences of symptoms and testing decisions**	COVID-19 testing criteria (continuous cough, temperature) are experienced as ambiguous	• Responsibility for the risk one poses to others, and moral duty to test when symptomatic or at increased risk of infection • Ethical tensions between moral responsibility to test versus uncertainty about symptoms and/or personal/social/economic costs of testing
	Concerns about exposure and risk	
**Accessing tests**	Booking systems can be arduous to navigate	• Frustration with government over challenges of accessing tests • Individual responsibility for not spreading infection through travel (e.g., public transport) • Obligations to employer
	Challenges getting to a testing centre	
	Taking time off work	
**Sample collection**	Experiences of physical discomfort	• Duty to get oneself or one’s child tested • Parental responsibility for children’s physical and emotional wellbeing • Frustration with government over hidden challenges of testing
	Difficulties interpreting instructions and guidance	
	Doubts about accuracy of self-swabbing method	
**Waiting for, receiving, interpreting, and acting on results**	Ambivalence about self-isolating while awaiting a test result	• Lack of recognition by government of personal costs of testing and isolation • Ethical obligation to isolate balanced with personal circumstances and/or social obligations to others • Responsibility for self-diagnosis
	Negative test result enables a return to work, and social obligations to be fulfilled	
	People question accuracy of testing when test results do not align with their diagnostic suspicions	
	Willingness to self-isolate following a positive test result, despite anticipated challenges	

### Perceived benefits of testing

Participants identified multiple benefits of testing, including for the person undergoing testing, for immediate members of their social network (household members, friends, and family) and for the wider community. Illustrative quotes for these and other thematic findings can be found in the Supplementary Materials (Tables S1, S2, S3, S4, S5, and S6).

Participants valued the information that testing provided about their personal health status, even when this information did not inform medical decision making. This was especially important for people with underlying health conditions and for those who perceived themselves to be at higher risk of exposure.

I suffer from anxiety, which I didn’t actually before the pandemic. I suppose on a personal level, having a test and having a negative result reassured me […] It definitely gave a sense of reassurance and kind of relief that we weren’t spreading it either (male, 28, public sector worker, participant 30).

It’s about knowing whether you’ve had it. I was really unwell with COVID and I would hate to ever have to go through that again. So yes, there would be assurances that I had the antibodies that hopefully if I was exposed to it again, I wouldn’t get it again (female, 50, officer worker, participant 88).

Another common personal benefit related to perceptions of social stigma around symptoms, and the desire to avoid moral judgement in interactions with others at school or in the workplace.

I knew it was just a cold. I was 1000% sure it was negative but because nursery needs the test because of COVID, and so I had to do this […] it was a new-ish continuous cough. It was definitely a cold but I can’t prove it’s not COVID without a test (female, 33, university lecturer, participant 51).

Participants saw members of their immediate social network, including household members, family, friends, and colleagues, as important beneficiaries of testing, especially in terms of potential asymptomatic transmission:

Because you might actually feel fine, you could be asymptomatic, but that’s always the danger … you could be passing it on to your friends and family without realising (female, 55, charity officer, participant 03).

Participants often felt particular concern about the safety and wellbeing of persons viewed as vulnerable to COVID-19, such as elderly parents or those with underlying medical conditions.

I’m generally quite healthy so I’d probably be alright, but my husband has had a heart attack. It was years ago, family history, so he’s obviously more of that situation. Me personally I’m not that – but I think I’d want to make sure that I don’t pass it on (female, 36, financial worker, participant 44).

Last, participants had a strong sense of the benefits of testing to the ‘community’ or ‘society’. This included awareness of the public health value of testing to the overall reduction of transmission, and contribution of testing data to disease surveillance, public health policy, and government planning.

There’s a sense of responsibility as well to everyone else around you, which I was quite happy to take home […] I think responsibility lies on the state to kind of encourage people to understand there is a sacrifice involved […] That’s collecting information that could be really, really useful to medical officers and to clinicians and to virologists (male, 28, public sector worker, participant 30).

### Expectations of testing provision and providers

Many participants framed their expectations of who should provide testing, who should bear its cost, and what testing services should look like in terms of a social contract between state and society. Several participants viewed the accessibility of the testing system as an indicator of the government’s competence, concern for the welfare of its citizens, and/or understanding of people’s everyday lives and constraints. Willingness to contribute to Test and Protect was often accompanied by the expectation that the government would provide the means and support for people to participate:

I think you can put a moral demand on the government, that states they actually have a duty to provide this for the people in the country. And if they’re not, then they’re really failing in one of their most basic duties (male, 30, student, participant 68).

In turn, participants understood that they were obliged to access testing when they met the testing criteria. This was often expressed in terms of the need for social ‘solidarity’ and a ‘collective’ response to the pandemic.

I kind of felt there was a bit of a duty upon ourselves to go and be tested because if we did have it, it’s better to know so we don’t spread it (male, 28, public sector worker, participant 30).

However, many also expressed reservations that they should only seek testing when necessary, to avoid wasting scarce resources.

I just wish we hadn’t done it. I felt a bit conflicted about even going […] I don’t like to think that we’re wasting resources, I know this is expensive (male, 50, office worker, participant 92).

Many participants also expressed discontent with private companies profiting from testing; some described a lack of trust in private contractors to provide accessible, functioning, and accurate testing services, or to use their data responsibly, expressing a preference that these services be provided through the NHS.

I think you would feel better if it’s being managed by the NHS; I think it’s more likely to be trusted. If you’re saying it’s being managed by an external company, a private company, I think that would be different. People may then think, a private company, it’s financially related, you know, that’s why you would worry about what would they do with the data (female, 50, office worker, participant 88).

I just think in the UK there’s just much more of that corporate influence and even just the whole healthcare system if they’ve been run as public or private partnerships and like social care partnerships. All this stuff that’s free as far as I understand it, it seems to just be less of a public good. My understanding is that there’s a lot more point at which there’s people making money basically. So that just makes me suspicious and I think that would be the main reason why I would trust it less (male, 30, student, participant 68).

### Experiences of symptoms and testing decisions

Participants did not always feel equipped to interpret symptoms and make a clinical judgement about whether they met the government’s testing criteria. The symptoms ‘continuous cough’ and ‘fever’ were experienced as ambiguous, especially in children. Participants often combined symptom interpretation with other information, such as their perceived levels of exposure (e.g., exposure at work or with reference to data on local case numbers); perceptions of their personal risk of severe disease; other people’s stories about testing; and media reports (e.g., concerning asymptomatic spreaders). ‘It was definitely a cold, but I can’t prove it’s not COVID without a test’ one respondent (female, 33, lecturer, Participant 51) told us.

Uncertainty over symptom identification opened up space for ethical uncertainty and deliberation over test-seeking, frequently entailing negotiation with household or family members. In the case of children, decision making often unfolded along gendered lines:

I chatted to my husband and yes, he kind of thought maybe it was a bit unnecessary, but I managed to persuade him that yes, [to get our son tested] it was the thing to do (female, 52, public sector advisor, participant 101).

In some cases, people reported waiting to see if symptoms persisted before self-isolating themselves and/or their children and booking a test. However, most participants who experienced doubts about eligibility, and even those who felt sure they or their children did not have COVID-19, still ultimately booked a test ‘to be on the safe side’.

We probably should have gone sooner because we just thought she had a cold. But then the cough wouldn’t go […] We ignored the cold certainly in the youngest [child] and their symptoms for days. I’d never have kept her off [school] for that normally, but we did (male, 50, officer worker, participant 92).

### Accessing testing

While accessing tests was a smooth process for many, other participants described encountering booking system errors, inconvenient test centre locations, a lack of availability in their area, and/or difficulties in negotiating COVID-safe transport. This work was often taken on by women and was experienced as disruptive to their everyday routines. Despite efforts to comply with guidance and high motivation to ‘do the right thing’, many participants expressed discontent with the effort involved in accessing a COVID-19 test, and the constraints that the system imposed on their capacity to act ethically and responsibly:

So, we would have had to take the bus [to the testing centre] and obviously because it was flaring up, I was thinking, “Okay, we don’t want to do that, because that’s irresponsible” (female, 19, student, participant 64).

There was this strange dynamic of actually needing to see people because we had these responsibilities to these neighbours, but not being able to because we couldn’t get a test and we just didn’t know if we were all dangerous (...) it made the fact that we couldn’t get a test feel quite an issue at the time, because we did have these responsibilities (female, 42, youth worker, participant 71).

Several interviewees experienced difficulty in negotiating time off work to go to the testing centre or to transport a dependent there. These challenges were often linked to the perception that they had been let down by government, and that politicians were ‘out of touch’ with their personal circumstances.

[My partner] and myself both had to take time off work to get tested and it ended up being almost half a day between the journey there and getting the testing and coming back and everything else, so it took a big chunk out of the day (male, 42, third sector manager, participant 72)

If you want me to be able to do a job in a safe way, then you need to provide the resources for that to be possible (male, 30, student, participant 68).

### Sample collection

Participants regularly described intense discomfort when using the nasopharyngeal swab tests. Formal guidelines presented the self-test as straightforward, but participants did not always find the instructions easy to follow and feared that they had collected the sample incorrectly. Swabbing younger children and persons with special needs presented an emotional and physical challenge for which parents felt under-prepared. Most first-hand stories of testing children were narrated by female participants in the study.

There was a drawing where the instructions made you feel like you should be able to do this happy thing and make a funny face and give a toy or do something and it’s going to work. And I did all of those things with my first son and as soon as I started the test, he just freaked out (female, 33, university lecturer, participant 51).

Discontent with testing experiences was linked by several participants to a distrust of privately contracted testing services, and their lack of integration with the broader healthcare system. For example, some people expressed concerns about non-clinical staff performing tests.

It probably helps if it’s a health professional involved in that, it might give somebody some confidence that people know what they’re doing and it’s not just Joe Bloggs poking you with a stick. It might help from some public confidence point of view (female, 39, NHS staff, participant 09).

### Waiting for, receiving, interpreting, and acting on results

Some participants found the period of self-isolation while awaiting results emotionally and practically challenging. Consequences of testing (even when an individual tested negative) often meant reneging on other moral and social duties, in particular the care of family members within and beyond the household, and duties towards employers and colleagues, which also had financial consequences:

The ramifications of that false-positive test were awful last week. Our employer told us that we might not get paid (female, 53, carer, participant 36).

A negative result provided reassurance about personal health in addition to relief that social interactions at home and at work could resume. But test results were not necessarily taken at face value and were often interpreted in the context of diagnostic suspicion (e.g., based on perceived risk of exposure in the workplace, knowledge of local prevalence, observations of other people’s behaviour, close contact with a confirmed case, and/or combination or severity of symptoms). Receiving a test result that conflicted with those suspicions raised doubts for some people about the quality and accuracy of tests, or the competencies of those administering them.

They [test results] both came back negative, which was really very puzzling to us. It’s still very puzzling to me, like how we came into direct contact with somebody who stayed at the flat, and not to get it seemed really quite wild’ (female, 32, student, participant 16).

A little bit of me was worried because it was self-administered […] you know, the level of false negatives of the self-administered test, so that’s what I was trying to avoid […] I’d seen how badly [my daughter] was gagging, trying to find the back tonsils and then trying to do the nasal bit. So, it’s a possibility she didn’t do it properly. I was 85% sure that was correct (female, 46, NHS staff, participant 85).

All participants indicated their willingness to follow government guidelines to self-isolate following a positive test or identification as a close contact, with some referring to a ‘moral duty’ to do so. However, participants indicated various challenges associated with self-isolation, including food provision, children’s wellbeing, and pet care.

It is on my mind quite a lot, that there is a potential that at some point, the test and trace are going to call me to say, “As a household you need to isolate and if you develop symptoms, get a test”. Now my concern is obviously about I really don’t want that for my girls, because they’ve lost so much school already this year, I don’t want them to lose any more school. I think they are falling so behind. So that would really – it would make me anxious, you know, having to self-isolate. But at the same time, you have a responsibility for the health and wellbeing of everybody. So, I absolutely would do that (female, 50, officer worker, participant 88).

### Pandemic time

Throughout the data collection period, people’s views on COVID-19 testing and experiences of COVID-19 testing that are reported above were continually refracted through pandemic events, including changes in the epidemiology of the disease, scientific and technical developments, public events that placed particular pressure on the Test and Protect system, such as the reopening of schools and universities, and changes to government policies, such as the imposition or easing of lockdown rules. Concerns about access to testing and, conversely, about wasting testing resources reported above, were both prominent at the beginning of the data collection period when test shortages were widely reported in the media [62–67]. Moral statements about who should be able to access testing and who should pay for it became more prominent during the summer months of 2020 when many people in Scotland were planning holiday travel.

I think if you want to go on holiday and you have the option to pay for a test, that’s fine […] I think there’s a responsibility on [airlines] also to offer testing as an option. I think airlines and travellers themselves definitely have a responsibility to provide solutions (male, 28, public sector worker, participant 30).

Frustration with the booking system, and the government response in general, were more prevalent during the period when schools reopened and the Test and Protect system struggled to sustain services under intense demand. The ethical dilemmas of testing children reported above were also especially prominent in this period. Other reported social and ethical considerations remained consistently prominent throughout the period of data collection, including a concern with civic responsibility and social solidarity, and a concern to protect one’s own health and the health of friends and family.

## Discussion

This study contributes to understandings of the social and ethical aspects of COVID-19 testing in the early months of the pandemic. At the time of data collection, there was widespread scientific uncertainty over the virulence of the virus and its dominant modes of transmission, hospital admissions were high, and fear of the virus was widespread. For much of this period, the Scottish public was living under strict lockdown measures that were unprecedented in their restriction of personal freedoms and were justified by epidemiological models showing a potentially devastating loss of life. The Scottish and UK government testing policies were also the subject of sustained public controversy during this period, relating to delays in establishing community-based testing, widespread test shortages, and the public financial cost of testing programmes, especially in relation to private sector contracts [68, 69].

Our research shows that, in the context of a public health emergency, members of the Scottish public are overwhelmingly willing to undergo medical testing, even where this has few personal benefits in terms of medical treatment and entails significant social and economic costs. This finding aligns with other UK research that shows high levels of self-reported willingness to comply with guidelines during the pandemic [23], including after vaccination [70].

We found that people’s narratives of testing and their testing decisions and practices were shaped by ethical considerations at every stage of the testing process [55]. People were primarily motivated to seek testing by a sense of civic duty to protect ‘society’, by a sense of solidarity with others [26, 29] and by ethical obligations towards specific individuals within their social network. Changing social norms and expectations around symptomatic behaviours also had an influence, with evidence of an emerging testing ‘etiquette’ in the context of visible cold symptoms [71]. Trust in government and the testing system were found to be important positive enablers of participation [22].

While participants were willing to test in principle, many also reported experiencing conflicting ethical obligations in practice, such as when isolation requirements obligated them to let down friends, family, or employers. These findings corroborate research showing that lower compliance with testing guidelines is associated with caring responsibilities for relatives and friends, and work outside the home [70]. The dilemmas or ‘moral breakdowns’ [49] that these conflicting obligations created were exacerbated by ambiguities in the testing criteria, and in some cases led to delays in seeking testing in what others have called a ‘wait and see’ approach [20]. Echoing findings from other social research on COVID-19 testing, people also described frustration at challenges in accessing and undergoing testing, including not knowing where to go to book a test or get the sample taken, or concerns about eligibility [7], transportation, and the physical discomfort of testing [10–13]. Our findings show that these practical impediments to testing also had an ethical dimension since they often caused participants to weigh up the moral implications of different avenues of action, such as whether to test one’s children if this causes them significant distress. Such dilemmas revealed participants’ competing responsibilities, obligations and struggles to be a good parent, friend, colleague or citizen [43]. COVID-19 testing is therefore a multi-faceted ethical process that implicates social relationships at multiple scales, and cannot only be understood in terms of a conflict between individual interest and the public good [42, 72].

The disconnect between the apparently straightforward testing process as presented by government guidance [73]—whether regarding criteria for testing, booking systems, self-test instructions, or self-isolation rules—and the everyday challenges and dilemmas that arose when those guidelines met individual circumstances created space for uncertainty and moral ambiguity. We argue that people’s attempts to deal with this gap between expectations and experienced reality can be understood as a hidden ethical burden of testing. Ethical burdens involve negotiations, evaluations, time and affective labour put into weighing up the best course of action to take under the circumstances, and reflecting on the future consequences of such a decision [43]. While the economic burden of testing in terms of the potential costs of isolation has been widely discussed [74–76], neither the social costs of testing, nor the everyday emotional and cognitive ‘work’ of ethical reasoning and decision making are widely acknowledged. This is despite social and ethical issues being at the forefront of the testing experience for members of the public [4, 9, 42]. We therefore argue that the burden of testing needs to be understood as practical, economic *and* ethical and that navigating the ethics of COVID-19 testing can be considered a hidden form of public health and epidemic response work [50, 51].

Even when the burden of testing does not prevent people from following guidelines, it should still be a priority for governments to identify and reduce the burden of policy interventions where this is possible. Not only is this a moral obligation of the state, it is also a question of socioeconomic equality. The higher number of women compared to men who enrolled in this study, together with the fact that first-hand stories of testing children most often came from female participants, suggests the distribution of the testing burden may be gendered. This finding confirms existing research on the gendered impacts of COVID-19 [77, 78] and indicates the need for further research in this area. A limitation of this study was that we did not focus on socioeconomically deprived or marginalised groups, which research has shown to be disproportionately affected by pandemic policies [79–83], and we recommend that further research is carried out on the relationship between testing burdens and socioeconomic inequality more broadly.

Several further limitations of the study affect the generalisability of the findings, including the use of online recruitment and interview methods and the dependence on self-reporting. Despite targeted recruitment methods and purposive sampling, we did not enrol substantial numbers of participants aged 80 and over. Only two participants tested positive prior to being interviewed, which limited our insight into people’s experience of the consequences of testing positive. In line with conventions for anthropologically informed qualitative research, validity, rather than representativity, was the main quality parameter for this study [50, 84].

## Conclusion

This study contributes to understanding how people perceived and experienced COVID-19 testing policies in Scotland during the early stages of the pandemic. Our findings confirm existing evidence that people are largely accepting of COVID-19 testing technologies and guidelines. They also show a relationship between this acceptance and people’s understandings of their moral obligations and responsibilities towards others. The ethical dilemmas involved in testing were found to be more complex than a simple opposition between personal self-interest and the public good would suggest. People’s views and engagements with testing were shaped by obligations and relationships at multiple social scales, including responsibility for one’s own health and wellbeing, obligations to others within one’s immediate social network, and a civic duty to ‘community’ or ‘society’.

We found that COVID-19 testing often entailed social, economic, and practical costs for individuals, which in some cases resulted in competing obligations and ethical dilemmas. Programme failures to acknowledge and mitigate competing obligations, the challenges involved in accessing testing, and the social and economic costs that testing entailed for people, generated uncertainty and left some people feeling that the government was ‘out of touch’ with everyday lives. Nonetheless, people’s sense of moral obligation to follow guidelines, and in particular their concern to protect other people from COVID-19 and to contribute to a wider community response, usually outweighed other considerations for participants. This demonstrates the important role played by moral judgements about what is ‘the right thing to do’ in shaping responses to public health guidelines, an area that does not always receive attention in traditional public health studies of compliance and adherence.

However, we know that moral expectations and judgements change through time in relation to the specific cultural, social and historical contexts within which people find themselves [85]. Developments in the science, politics and epidemiology of the pandemic since this study was conducted are therefore likely to have led to changes in people’s understandings, perceptions and experiences of COVID-19 testing. For example changes in government guidelines and testing provision, such as the introduction (and later withdrawal) of free home-testing with lateral flow tests [86] might change people’s perception of their obligations to the state; the re-opening of the economy [75] might change people’s perceptions of social solidarity and their obligations to the community; and mass vaccination [87] and the emergence of new variants [76] might change people’s perceptions of risk and therefore the weight of ethical obligations to test so as to protect others. This study therefore provides insight to the ethical dimensions of COVID-19 testing at a particular historical moment in the pandemic in Scotland, and further research is needed to understand how those ethical dimensions might have changed over time.

Understanding the social and ethical expectations that voluntary COVID-19 testing places on people across the testing process, and acknowledging the challenges that people face when incorporating testing into their everyday lives are crucial to ensuring that COVID-19 testing does not impose unnecessary burdens on people. Consideration of whether and how testing policies disproportionately affect certain groups, for example women with children, is also essential to the equitable design of medical testing programmes in the future. Our findings point to the need for further research into the relationship between the ethical burden of testing and the uptake of voluntary testing programmes over the course of the pandemic.

## Supplementary Information

**Additional file 1:** **TableS1.** Perceived benefits of testing and motivations to participate in test andprotect. **TableS2.** Expectations of, and trust in, testing provision and providers. **TableS3.** Experiences of symptoms and decisions to test. **TableS4.** Accessing tests. **TableS5.** Sample collection. **Table S6.** Waiting for, receiving, interpreting and acting onresults.

## Data Availability

The datasets generated and/or analysed during the current study are not publicly available due to ethical constraints related to the sensitive nature of the interview material, but are available from the corresponding author on reasonable request.

## Abbreviations

PCR: Polymerase chain reaction

## References

[1] Iacobucci G (2020). Covid-19: What is the UK’s testing strategy?. BMJ.

[2] Freedman L (2020). Strategy for a Pandemic: The UK and COVID-19. Survival (Lond).

[3] World Health Organization. WHO Director-General’s opening remarks at the media briefing on COVID-19 - 16 March 2020. 2020. Accessed 2022 Feb 16. Available from: https://www.who.int/director-general/speeches/detail/who-director-general-s-opening-remarks-at-the-media-briefing-on-covid-19---16-march-2020.

[4] Bevan I, Baxter MS, Stagg HR, Street A, van Bunnik B (2021). Knowledge, attitudes, and behavior related to COVID-19 testing: a rapid scoping review. Diagnostics.

[5] Clipman SJ, Wesolowski A, Mehta SH, Agarwal S, Cobey SE, Solomon SS (2021). Sars-cov-2 testing in florida, illinois, and maryland: access and barriers. Top Antivir Med.

[6] Graham MS, May A, Varsavsky T, Sudre CH, Murray B, Kläser K, et al. Knowledge barriers in the symptomatic-COVID-19 testing programme in the UK: an observational study. medRxiv. 2021;2021.03.16.21253719.

[7] Mcelfish PA, Purvis R, James LP, Willis DE (2021). Perceived barriers to COVID-19 testing. Int J Environ Res Public Health.

[8] Zimba R, Kulkarni S, Berry A, You W, Mirzayi C, Westmoreland D (2020). SARS-CoV-2 testing service preferences of adults in the United States: Discrete choice experiment. JMIR Public Heal Surveill.

[9] Martindale AM, Pilbeam C, Mableson H, Tonkin-Crine S, Atkinson P, Borek A (2021). Perspectives on COVID-19 testing policies and practices: a qualitative study with scientific advisors and NHS health care workers in England. BMC Public Health.

[10] Hofschulte-Beck SL, Hickman SE, Blackburn JL, Mack LM, Unroe KT (2021). Attitudes and experiences of frontline nursing home staff toward coronavirus testing. J Am Med Dir Assoc.

[11] Kawamura H, Orisaka M, Yoshida Y (2021). Mentality of pregnant women and obstetric healthcare workers about prenatal SARS-CoV-2 testing: A regional survey over the first wave of the COVID-19 pandemic in Japan. J Obstet Gynaecol Res.

[12] Kernberg A, Kelly J, Nazeer S, Russell S, Tuuli M, Stout MJ (2020). Universal severe acute respiratory syndrome coronavirus 2 (SARS-COV-2) testing uptake in the labor and delivery unit: implications for health equity. Obstet Gynecol.

[13] Denford S, Martin AF, Love N, Ready D, Oliver I, Amlôt R (2021). Engagement with daily testing instead of self-isolating in contacts of confirmed cases of SARS-CoV-2: a qualitative analysis. Front Public Heal.

[14] Asgary A, Cojocaru MG, Najafabadi MM, Wu J (2021). Simulating preventative testing of SARS- CoV-2 in schools: policy implications. BMCPublic Heal.

[15] Smith LE, Potts HWW, Amlȏt R, Fear NT, Michie S, James Rubin G. Adherence to the test, trace and isolate system: Results from a time series of 21 nationally representative surveys in the UK (the COVID-19 Rapid Survey of Adherence to Interventions and Responses [CORSAIR] study). medRxiv. 2020;1–47.

[16] Fabella FE. Factors Affecting Willingness to be Tested for COVID-19. SSRN Electron J. 2020;

[17] Khalifa AM, Alshammari AF, Alrimali AM, Alshammari RA (2021). Willingness to Test for COVID-19: A Cross-Sectional Study on the Population in the Ha’il Region. KSA J Pharm Res Int.

[18] McGowan CR, Hellman N, Chowdhury S, Mannan A, Newell K, Cummings R (2020). COVID-19 testing acceptability and uptake amongst the Rohingya and host community in Camp 21, Teknaf. Bangladesh Confl Health.

[19] Ortega-Paredes D, Zurita J, Zurita C, Leoro-Garzón P, Leoro-Monroy G, Larrea-álvarez CM (2021). An on-line cross-sectional questionnaire to assess knowledge of COVID-19 pandemic among citizens tested for the SARS-CoV-2 virus in Quito and Ibarra, Ecuador. Int J Environ Res Public Health.

[20] Mowbray F, Woodland L, Smith LE, Amlôt R, Rubin GJ (2021). Is my cough a cold or covid? a qualitative study of COVID-19 symptom recognition and attitudes toward testing in the UK. Front Public Heal.

[21] Ferree KE, Harris AS, Dulani B, Kao K, Lust E, Metheney E (2021). Stigma, trust, and procedural integrity: Covid-19 testing in Malawi. World Dev.

[22] Bollyky TJ, Hulland EN, Barber RM, Collins JK, Kiernan S, Moses M (2022). Pandemic preparedness and COVID-19: an exploratory analysis of infection and fatality rates, and contextual factors associated with preparedness in 177 countries, from Jan 1, 2020, to Sept 30, 2021. Lancet.

[23] Atchison C, Pristerà P, Cooper E, Papageorgiou V, Redd R, Piggin M (2021). Usability and acceptability of home-based self-testing for severe acute respiratory syndrome coronavirus 2 (SARS-CoV-2) antibodies for population surveillance. Clin Infect Dis.

[24] Bender WR, Srinivas S, Coutifaris P, Acker A, Hirshberg A (2020). The psychological experience of obstetric patients and health care workers after implementation of universal SARS-CoV-2 testing. Am J Perinatol.

[25] Blake H, Corner J, Cirelli C, Hassard J, Briggs L, Daly JM (2021). Perceptions and experiences of the university of nottingham pilot sars-cov-2 asymptomatic testing service: a mixed-methods study. Int J Environ Res Public Health.

[26] Christensen SW, Dagyaran I, Bernild C, Missel M, Berg SK (2021). Testing for COVID-19 regulates behavior in the general population: A qualitative study of experiences of awaiting test result for COVID-19. Scand J Public Health.

[27] De Camargo C. ‘It’s tough shit, basically, that you’re all gonna get it’’: UK virus testing and police officer anxieties of contracting COVID-19.’ Polic Soc. 2021;0(0):1–17.

[28] Lecouturier  J, Kelly  MP, Graham  F, Meyer  C, Tang  MY, Goffe  L (2021). Public understanding of COVID-19 antibody testing and test results: a qualitative study conducted in the U.K. early in the pandemic. Soc Sci Med.

[29] Missel M, Bernild C, Dagyaran I, Christensen SW, Berg SK (2020). A stoic and altruistic orientation towards their work: a qualitative study of healthcare professionals’ experiences of awaiting a COVID-19 test result. BMC Health Serv Res.

[30] Bryman, A. Social Research Methods: Fifth Edition. Oxford University Press; 2015.

[31] Engel N, Davids M, Blankvoort N, Dheda K, Pant Pai N, Pai M (2017). Making HIV testing work at the point of care in South Africa: a qualitative study of diagnostic practices. BMC Health Serv Res.

[32] Yellapa V, Devadasan N, Krumeich A, Pai NP, Vadnais C, Pai M (2017). How patients navigate the diagnostic ecosystem in a fragmented health system: a qualitative study from India. Glob Health Action.

[33] Vernooij E, Koker F, Street A. Responsibility, repair and care in Sierra Leone’s health system. Soc Sci Med. 2021; 10.1016/j.socscimed.2021.114260 34315638

[34] Birenbaum-Carmeli D, Inhorn MC, editors. Assisting reproduction, testing genes: Global encounters with the new biotechnologies. Berghan Books; 2009.

[35] Beisel U, Umlauf R, Hutchinson E, Chandler CIR (2016). The complexities of simple technologies: re-imagining the role of rapid diagnostic tests in malaria control efforts. Malar J.

[36] Latimer J (2007). Diagnosis, dysmorphology, and the family: knowledge, motility, choice. Med Anthropol Cross Cult Stud Heal Illn.

[37] Lupton D (2012). “Precious cargo”: Foetal subjects, risk and reproductive citizenship. Crit Public Health.

[38] Rapp R. Testing women, testing the fetus: The social impact of amniocentesis in America. Routledge; 2004.

[39] Konrad M (2003). Predictive genetic testing and the making of the pre-symptomatic person: Prognostic moralities amongst Huntington’s-affected families. Anthropol Med.

[40] Jutel A, Nettleton S (2011). Towards a sociology of diagnosis: Reflections and opportunities. Soc Sci Med.

[41] Timmermans S, Buchbinder M (2010). Patients-in-waiting: Living between sickness and health in the genomics era. J Health Soc Behav.

[42] Street A, Kelly AH (2021). Introduction: medical testing. Diagnosis and Value Med Anthropol Theory.

[43] Mattingly C. Moral laboratories: Family peril and the struggle for a good life. University of California Press; 2014.

[44] Robbins J (2013). Beyond the suffering subject: Toward an anthropology of the good. J R Anthropol Inst.

[45] Das V (2007). Life and words: Violence and the descent into the ordinary.

[46] Faubion JD (2001). The Ethics of Kinship: Ethnographic Inquiries.

[47] Lambek M (2010). Ordinary ethics: Anthropology, language, and action.

[48] For LJ, Freedom AAOEA (2002). J R Anthropol Inst.

[49] Zigon J. Moral breakdown and anthropology of moralities. Anthropol Theory. 7:131–50.

[50] Gammeltoft TM, Diệu BTH, Dung VTK, Thị ÁN, Hiếu LM (2022). Everyday disease diplomacy : an ethnographic study of diabetes self-care in Vietnam. BMC Public Health.

[51] Lee SJ, Vernooij E, Enria L, Kelly AH, Rogers J, Ansumana R (2022). Human preparedness: relational infrastructures and medical countermeasures in Sierra Leone. Glob Public Health.

[52] Steel MK, Stevenson K, Evans J, McCormick C, Willocks D, McCallum L (2020). Coronavirus disease (COVID-19) community testing team in Scotland: a 14-day review, 6 to 20 February 2020. Euro Surveill.

[53] Public Health Scotland. COVID-19 statistical reports.

[54] Scotland PH. COVID-19 Daily Dashboard, COVID-19 in Scotland. 2020.

[55] Mattingly C (1998). Healing dramas and clinical plots: The narrative structure of experience.

[56] Squire C, Andrews M, Squire C, Tamboukou M (2008). Experience-centred and culturally-oriented approaches to narrative. Doing narrative research.

[57] Braun V, Clarke V. Thematic analysis. APA Handb Res Methods Psychol. 2012;2.

[58] Esin C, Fathi M, Squire C (2014). Narrative analysis: the constructionist approach. SAGE Handb Qual Data Anal.

[59] Squire C. HIV in South Africa: Talking about the big thing. HIV South Africa Talk About Big Thing. 2007;1–229.

[60] Mattingly C (2013). Moral selves and moral scenes: narrative experiments in everyday life. Ethnos.

[61] Public Health Scotland. COVID-19 Daily Dashboard, COVID-19 in Scotland 6 July 2020. 2020. Available from https://public.tableau.com/app/profile/phs.covid.19/viz/COVID-19DailyDashboard_15960160643010/Dailyupdate. Accessed 2022 July 21.

[62] Pollock AM, Roderick P, Cheng KK, Pankhania B. Covid-19: why is the UK government ignoring WHO’s advice? BMJ. 2020;368. 10.1136/bmj.m1284 32229543

[63] Horton R (2020). Offline: COVID-19 and the NHS—“a national scandal”. Lancet.

[64] Horton R (2020). COVID-19 testing delays and pathology services in the UK. Lancet.

[65] Mason R, Campbell D. Pressure on Johnson as UK’s daily coronavirus testing target missed again. The Guardian. 2020. Accessed 2022 Feb 16. Available from: https://www.theguardian.com/world/2020/may/06/boris-johnson-misses-coronavirus-testing-target-for-fourth-day-in-a-row.

[66] Perraudin F, Duncan P. Britain’s coronavirus testing scandal: a timeline of mixed messages. The Guardian. 2020. Accessed 2022 Feb 16. Available from: https://www.theguardian.com/politics/2020/apr/03/coronavirus-testing-in-uk-timeline-of-ministers-mixed-messages

[67] ITV. Coronavirus testing in UK not ramped up as quickly as it should have been, Sir Patrick Vallance admits. 2020. Accessed 2022 Feb 16. Available from: https://www.itv.com/news/2020-04-13/coronavirus-testing-in-uk-not-ramped-up-as-quickly-as-it-should-have-been-government-s-chief-scientific-advisor-admits

[68] Wise J (2020). Covid-19: What’s going wrong with testing in the UK?. BMJ.

[69] Godlee F (2020). Covid 19: Where’s the strategy for testing?. BMJ.

[70] Wright AL, Sonin K, Driscoll J, Wilson J (2020). Poverty and economic dislocation reduce compliance with COVID-19 shelter-in-place protocols. J Econ Behav Organ.

[71] Alenichev A (2020). Ethics and etiquette in an emergency vaccine trial. The orchestration of compliance Glob Bioeth.

[72] Reicher S, Drury J (2021). Pandemic fatigue? How adherence to covid-19 regulations has been misrepresented and why it matters. BMJ.

[73] Carers of West Lothian. Test & Protect: A Step by Step Guide. 2021. Available from: https://carers-westlothian.com/test-and-protect/

[74] Bodas M, Peleg K (2020). Income assurances are a crucial factor in determining public compliance with self-isolation regulations during the COVID-19 outbreak – cohort study in Israel. Isr J Health Policy Res.

[75] Chung S-C, Marlow S, Tobias N, Alogna A, Alogna I, You S-L (2021). Lessons from countries implementing find, test, trace, isolation and support policies in the rapid response of the COVID-19 pandemic: a systematic review. BMJ Open.

[76] Cardwell K, O’Neill SM, Tyner B, Broderick N, O’Brien K, Smith SM (2022). A rapid review of measures to support people in isolation or quarantine during the Covid-19 pandemic and the effectiveness of such measures. Rev Med Virol.

[77] Smith J, Davies SE, Feng H, Gan CCR, Grépin KA, Harman S (2021). More than a public health crisis: A feminist political economic analysis of COVID-19. Glob Public Health.

[78] Zamberlan A, Gioachin F, Gritti D (2021). Work less, help out more? The persistence of gender inequality in housework and childcare during UK COVID-19. Res Soc Stratif Mobil.

[79] Vargas Hill R, Narayan A. Covid-19 and inequality: a review of the evidence on likely impact and policy options. Centre for Disaster Protection. 2020.

[80] Fisher J, Languilaire JC, Lawthom R, Nieuwenhuis R, Petts RJ, Runswick-Cole K (2020). Community, work, and family in times of COVID-19. Community, Work Fam.

[81] Hu Y (2020). Intersecting ethnic and native–migrant inequalities in the economic impact of the COVID-19 pandemic in the UK. Res Soc Stratif Mobil.

[82] Wildman J (2021). COVID-19 and income inequality in OECD countries. Eur J Heal Econ.

[83] Health TLP (2021). COVID-19—break the cycle of inequality. Lancet Public Heal.

[84] Leung L (2015). Validity, reliability, and generalizability in qualitative research. J Fam Med Prim Care.

[85] Zigon J, Throop CJ (2014). Moral experience: Introduction. Ethnos.

[86] Scottish Government. Regular rapid testing for everyone. 2021. Accessed 2022 Feb 16. Available from: https://www.gov.scot/news/regular-rapid-testing-for-everyone/

[87] Scottish Government. First COVID-19 vaccinations in Scotland take place. 2020. Accessed 2022 Feb 16. Available from: https://www.gov.scot/news/first-covid-19-vaccinations-in-scotland-take-place/

