# Supplementary material for: The hidden burden of medical testing: public views and experiences of COVID-19 testing as a social and ethical process
Source: BMC Public Health. 2022 Sep 30;22:1837. doi: 10.1186/s12889-022-14217-2 (PMC9524338; doi:10.1186/s12889-022-14217-2)
Supplement: Supplementary file 1 — Additional file 1: TableS1. Perceived benefits of testing and motivations to participate in test andprotect. TableS2. Expectations of, and trust in, testing provision and providers. TableS3. Experiences of symptoms and decisions to test. TableS4. Accessing tests. TableS5. Sample collection. Table S6. Waiting for, receiving, interpreting and acting onresults. [file 12889_2022_14217_MOESM1_ESM.docx]

# The Hidden Burden of Testing: Supplementary Data

### Table S1. Perceived benefits of testing and motivations to participate in Test and Protect

| Beneficiary | Benefit | Social and ethical considerations | Example quotes |
| --- | --- | --- | --- |
| For the person getting tested | Knowledge of personal COVID-19 status | Reassurance of own health | ‘It’s about knowing whether you’ve had it. I was really unwell with COVID and I would hate to ever have to go through that again. So yes, there would be assurances that I had the antibodies that hopefully if I was exposed to it again, I wouldn’t get it again’ (female, 50, officer worker, participant 88).  ‘I think for me it would remove the uncertainty. It’s really frustrating not knowing if you’ve had it or not. And it also takes away some of the fear, if you’ve had it without even realising you’ve had it, then it takes away all the fear of it’ (female, 36, private sector manager, participant 52).  ‘It’s about knowing whether you’ve had it. I was really unwell with COVID and I would hate to ever have to go through that again. So yes, there would be assurances that I had the antibodies that hopefully if I was exposed to it again, I wouldn’t get it again’ (female, 50, officer worker, participant 88). |
|  | Reassurance that one is living responsibly, by not infecting others | Ethical commitment to not harm others | ‘There was this strange dynamic of actually needing to see people because we had these responsibilities to these neighbours, but not being able to because we couldn’t get a test and we just didn’t know if we were all dangerous, basically’ (female, 42, youth worker, participant 71).  ‘I think it’s the not knowing, it’s the ‘not sure’, it’s the ‘is it just a cold and can I legitimately go out for a walk and be sure that I’m not going to infect somebody else or I’m not going to disperse it around’ or not?’ (female, 57, teacher, participant 15).  ‘Because you might actually feel fine, you could be asymptomatic, but that’s always the danger is that you could be passing it on to your friends and family without realising’ (female, 55, third sector manager, participant 03)  ‘Both my colleague and myself were really quite distressed at the thought that we may have passed on the virus’ (female, 53, private sector carer, participant 36). |
|  | Avoidance of social stigma | Concern about moral judgements of others | ‘It just felt like if you were at home waiting for a test, then it would have been bad to pop to Ikea on the way home from the test centre or whatever. It felt socially unacceptable, even if I didn’t think it was a genuine concern’ (female, 42, youth worker, participant 71).  ‘There’s no testing, it would have been just useful to know and it ended up being a bacterial infection, I think because it got better with antibiotics really quickly. So there was no testing. I think mostly my husband and I got coughs, like those types of bacterial cough, and the nurse on the line was like, “You don’t sound like you have COVID.” And I was trying to be like, “You don’t know that, you don’t know what a COVID cough sounds like.” But it gives you that false hope. And then it’s harder for me to argue with my husband about, “We need to stay inside, we need to take precautions, because the nurse told me I probably don’t have COVID.” But she doesn’t know that.’ (Female, 33, Lecturer, participant 51).  ‘I knew it was just a cold. I was 1000% sure it was negative but because nursery needs the test because of COVID, and so I had to do this […] it was a new-ish continuous cough. It was definitely a cold but I can’t prove it’s not COVID without a test’ (female, 33, university lecturer, participant 51). |
| For immediate social contacts | Keeps them safe | Obligations of care towards family/friends/colleagues | 'I’m generally quite healthy so I’d probably be alright, but my husband has had a heart attack. It was years ago, family history, so he’s obviously more of that situation. Me personally I’m not that – but I think I’d want to make sure that I don’t pass it on.' (Female, 36, financial worker, participant 44).  I feel that if I found I was tested positive, if I had anything arranged like visiting family or friends I would probably postpone that and just not put other people at risk. Because as I say, there are so few people I know that have actually had it, I wouldn’t want to put them at risk (Female, 51, art teacher, participant 17).  Because you might actually feel fine, you could be asymptomatic, but that’s always the danger … you could be passing it on to your friends and family without realising’ (female, 55, charity officer, participant 03).  'I’m quite a sociable person, I like to see people and go out and about and not be stuck in the house, so that was hard, but we did it, because that was the right thing to do.' (Female, 39, charity sector worker, participant 19)  ‘There was this strange dynamic of actually needing to see people because we had these responsibilities to these neighbours, but not being able to because we couldn’t get a test and we just didn’t know if we were all dangerous (...) it made the fact that we couldn’t get a test feel quite an issue at the time, because we did have these responsibilities. We were part of this community that was doing this particular thing and it meant that we had to step out of that. And, also, we didn’t know if we’d passed anything on to people who were really vulnerable, so that was quite a stressful few weeks'. (female, 42, youth worker, participant 71).  ‘I think after I had the result, I was definitely way more like at peace with the fact that I could just socialise. Because as well my nieces and nephews and my grandmother as well, just in general it just made me more calm that I wasn’t a potential vector for all of these family members and stuff. So, in that sense I think it did change my behaviour’ (male, 30, student, participant 68). |
| For the local/national/global community | Reduces levels of transmission in population  Contributes to disease surveillance and policy/planning | Responsibility to the wider community to reduce transmission/ contribute to a collective response/ have solidarity with others  Social contract with the state | ‘It gives reassurance in the safety and the sense of being tested. [There are] lots of differences, but just more of a sense of community, for lack of a better word, in Denmark. A more communal response in a way, in the sense that it felt a bit more, well, there is this massive problem and we’re trying to solve it together. [In Scotland] I haven’t really felt that same sense of cohesion’ (male, 30, student, participant 68).  I kind of felt there was a bit of a duty upon ourselves to go and be tested,  because if we did have it, it’s better to know so we don’t spread it’ (male, 28 public sector worker, participant 30).  'I had decided to order a test and keep him and my daughter off school (…) I chatted to my husband and yes, he kind of thought maybe it was a bit unnecessary, but I managed to persuade him that yes, it was the thing to do.’ (female, 52, public sector advisor, participant 101).  ‘The testing station was in Dundee and we were in Forfar. So being the responsible human beings we are, we were like, “No, we cannot do that because obviously we’ll just infect the people there” […] So we would have had to take the bus and obviously because it was flaring up, I was thinking, “Okay, we don’t want to do that, because that’s irresponsible’ (female, 19, student, participant 64).  ‘There’s a sense of responsibility as well to everyone else around you, which I was quite happy to take home […] I think responsibility lies on the state to kind of encourage people to understand there is a sacrifice involved […] That’s collecting information that could be really, really useful to medical officers and to clinicians and to virologists’ (male, 28, public sector worker, participant 30). |

### Table S2. Expectations of, and trust in, testing provision and providers

| **Provider** | **Findings** | **Social and ethical considerations** | **Example Quotes** |
| --- | --- | --- | --- |
| UK Government/Scottish Government | Expectations about the availability of testing.  Concerns about wasting tests | Social contract between government and citizens.  Obligation of government to provide testing and make it accessible.  Obligation of public to access testing.  Obligation of public not to waste resources | 'If people are not responsible enough then you have to use discipline. We are all in the same place, so we’ve got to be more disciplined with this (...) I wouldn’t feel controlled by that. I would feel safer. That is different, because after this, it could take a year or two years, but it would just finish, so it’s only for now. It’s not a big thing.' (female, 37, teacher, participant 54).  'I think at what point do you sacrifice your own kind of personal liberties? I think you can measure it (...) There will be the financial side of it, there will be a massive hit at some point. But it’s understanding that, you know, there is a sacrifice involved' (male, 28, public sector worker, participant 30).    ‘There was all a big shout that the UK government [was] going to be able to do 100,000 tests a day, and the Scottish government [was] going to be able to do 20,000 tests a day (…) I think generally the information about testing has been very mixed; I don’t think there has been clarity and, again, you’re always hearing about more and more messages of people trying to get tested and the system’s not working’ (male, 68, sports club staff, participant 81).  ‘I think it just gives you more clarity in terms of all kinds of planning as well, like what risks you’re willing to take, what risks you’re prepared to expose others to and things like that. I think it will just be easier to negotiate if there was easier availability of testing.’ (male, 30, student, participant 68).  ‘In Scotland, even from my own personal experience, there were no tests available, there was no help structure for anyone who had COVID to actually get the test. It just wasn’t possible, so […] a lot of the time [I] felt very alone and you always think your government or your country is trying to support you and trying to help in some way, and I never had that feeling […] So yes, I did not feel I could trust Scotland’ (female, 19, student, participant 64).  ‘I think you can put a moral demand on the government that states they actually have a duty to provide this for the people in the country. And if they’re not, then they’re really failing in one of their most basic duties’ (male, 30, student, participant 68).  ‘I don’t think it should just be “trust people”, but I think it should be easy. I think you should make it as easy as possible for people to do the right thing. You can’t put up insurmountable barriers and then say, “Why aren’t people taking responsibility?” It should be easy and expected to do the right thing and there should be support’ (female, 33, university lecturer, participant 51).  ‘As much as there is a testing capacity, 15,000, 18,000, I kind of felt there was a bit of a duty upon ourselves to go and be tested, because if we did have it, it’s better to know so we don’t spread it’ (male, 28 public sector worker, participant 30).  ‘I’d rather we were able to live a better, fuller life, and if you have to wear a mask, you wear a mask. If you have to have a COVID test done to go to X Y and Z, then so be it’ (female, 50, office worker, 50, participant 88).  ‘I would feel worried about wasting a test on myself if it was a possibility that someone else could use it more. Maybe someone who works with vulnerable people directly’ (male, 29, care home staff, participant 63).  ‘I don’t like to think that we’re wasting resources, I know this is expensive’ (male, 50, officer worker, participant 92) |
| Private providers | Concerns about testing, or testing data, being a commodity, rather than a public good.  Difference between ‘private’ and ‘public’ uses of tests by individuals. | Unethical to make profit from a public good.  Leisure uses of testing (e.g. holiday travel) entail personal responsibility for testing. | ‘I think you would feel better if it’s being managed by the NHS; I think it’s more likely to be trusted. If you’re saying it’s being managed by an external company, a private company, I think that would be different. People may then think, a private company, it’s financially related, you know, that’s why you would worry about what would they do with the data’ (female, 50, office worker, participant 88).  ‘I think the British government is not just incompetent, I think they’re dangerous and I think they’re putting profits over health, so that’s why I would think that if I knew the testing was being done by a private company, or I knew that the test and trace apps were just a way to harvest all our data and they’re not really interested people’s health and public health, that’s why I’d trust them less’ (female, 36, university lecturer, participant 53).  ‘I just think in the UK there’s just much more of that corporate influence and even just the whole healthcare system if they’ve been run as public or private partnerships and like social care partnerships. All this stuff that’s free as far as I understand it, it seems to just be less of a public good. My understanding is that there’s a lot more point at which there’s people making money basically. So that just makes me suspicious and I think that would be the main reason why I would trust it less’ (male, 30, student, participant 68).  ‘I’ll give private data away, as long as I know it’s secure and it’s helping other people, as I’m doing for you and I’ve done for other COVID studies. But I’m very, very uncomfortable about the likes of G4S and companies akin to Cambridge Analytica, who sell your data on getting hold of it’ (female, 56, retired, participant 10).  ‘I’d be wary of any testing that is done for financial gain. I don’t think necessarily people should be trying to profit off of this. I don’t know, I think it’s a bit dodgy and if it means that people aren’t able to afford testing that would really such’ (female, 22, sales assistant, participant 38).  ‘Because there has to be an inclusive process. You don’t want people who have got the money paying for fast-tracking when you’ve got a little care home that desperately needs to have the reassurance of having the tests done that can’t afford to pay for the fast-tracking’ (female, 55, third sector manager, participant 03).  ‘It should be the government. It’s health. So definitely the government should take full responsibility for the health of its citizens’ (female, 27, student, participant 31).  ‘I know they are spending a lot of money on furlough and stuff, but it’s more important that people are getting tested and they’re protecting lives than a lot of other things. Even if it involved budget deficit and stuff I would still be for it’ (female, 22, sales assistant, participant 38).  ‘I think if you want to go on holiday and you have the option to pay for a test, that’s fine […] I think there’s a responsibility on [airlines] also to offer testing as an option. I think airlines and travellers themselves definitely have a responsibility to provide solutions’ (male, 28, public sector worker, participant 30).  ‘I’m not totally against the idea of people being able to privately pay for tests, but I am not sure why anybody would because what I would prefer is that the government were providing testing for everybody who needed testing and that that was accessible for everybody who actually needed that service’ (female, 55, teacher, participant 37). |
| Employers | Concerns about the distribution of the cost of testing. | Responsibility of employers to protect employees | ‘If your work puts you at risk, then I think it’s probably a work cost if it’s a private business. But if you’re working within the NHS or schools, local government, I think that should be your employer basically’ (female, 55, therapist, participant 07).  ‘Some other firms that either aren’t doing so well or struggling financially, which a lot of firms are, not being able to afford [testing] and then feeling guilt or anything like that, or feeling like they have to pay for it for their staff. I think it makes sense for me personally that that’s been coordinated and paid for at a Scottish Government level with the resources of the Government’ (male, 37, private sector manager, participant 104).  ‘If you want me to be able to do a job in a safe way, then you need to provide the resources for that to be possible’ (male, 30, student, participant 68). |

### Table S3. Experiences of symptoms and decisions to test

| **Factors in decision to seek test** | **Findings** | **Social and ethical considerations** | **Example quotes** |
| --- | --- | --- | --- |
| Symptoms | Covid-19 testing criteria (continuous cough, temperature) is experienced as ambiguous. | Ethical tensions between moral responsibility to test versus uncertainty about symptoms and/or personal/social/economic costs of testing | ‘I still didn’t find it very clear. And sometimes kids will cough a bit at bed time but then wake up the next morning and they’re not coughing. I don’t know. It still feels like a grey area to me, the cough part. The temperature and fever [are] really clear, but the cough part is not. I think I’d just take their temperature and we’ve never needed to get a test for a temperature, but I think if you had a sustained high temperature for more than 20 minutes apart or something, that would probably be enough for me’ (female, 42, private sector manager, participant 76).  ‘We weren’t going to get [my husband] tested initially because his cough wasn’t ‘*continuous*’, it was sort of like, “Does he have a cough, does he not have a cough?” […] I think the only thing would be I would be a bit more cautious on what constitutes a new continuous cough. I really would like guidance around that. Like, if my kid is coughing because they have a runny nose, is that a COVID cough? Is that a cough that’s significant enough to get a COVID test for?’ (female, 33, university lecturer, participant 51).  ‘I wasn’t going to get tested because I thought you needed two symptoms and I just had fever, but my wife said no even with one symptom you should go, so I said OK’ (male, 37, private sector manager, participant 104).  ‘Covid alters how you experience your own body. I wouldn’t know if my symptoms were ‘abnormal’ enough to fit, because my body lies outside of the range for ‘normal’ anyway’ (female, 32, student, participant 16).  ‘It became very apparent that this was definitely COVID. You know, the barking, the hacking, dry cough that she had and then the temperature, the terrible, terrible headache as well. So, I think – they didn’t get tested, no, but they absolutely had it. They definitely did’ (office worker, 50, female, participant 88).  ‘If it’s during term time, so while [my son] is at school, I think we would probably follow the recommended guidance, just to be on the safe side, because it’s such a big pool of infection otherwise. But if it’s during the holidays, we’d probably be more likely to wait a few days to see how it developed before getting tested’ (male, 42, third sector manager, participant 72).  'I was pretty sure my daughter just had a cold, because she seemed so much like she had earlier in the year. But obviously the symptoms were such that you kind of feel like you absolutely have to do your duty, and certainly not spread it to other bits of the NHS. So, I was really sure she didn’t have it but it was just the pressure of having to do the right thing, obviously (...) There was no way I’d have driven that distance if I could have just waited a bit longer, got the test, done it in the house. So, it was just trying to do the right thing and not get a false negative there.'  (Female, 46, NHS staff, participant 85). |
| Contact with others, local case data | Concerns about exposure and risk | Responsibility for the risk one poses to others, and moral duty to test when symptomatic, or at increased risk of infection. | ‘I would do things differently if it [cases] started to grow. We’d go back to shopping delivered or my wife wouldn’t let me go to the shop, even in a face mask and what have you. I think it would make you more aware, I think it would make everybody a little more sensitive, I think it would make them behave better’ (male, 68, sports club staff, participant 81).  ‘I was pretty sure my daughter just had a cold. But obviously the symptoms were such that you kind of feel like you absolutely have to do your duty, and certainly not spread it to other bits of the NHS. So, I was really sure she didn’t have it but it was just the pressure of having to do the right thing, obviously’ (female, 46, NHS staff, participant 85).  'When we were in lockdown, we were doing it every day, we were looking at the stats […] I do think it should be shared more routinely within communities about their own community because it might make the behaviours improve*.* There are a lot of people who I think would want to know, and help them make informed decisions about, “Will I go to the pub this weekend or will I not? Or will I go to the restaurant? Is there any need for me to do that?” It forces you, puts you into a lockdown position. People might make different choices in life to try and prevent the spread of the disease. I just think if the data was readily shared, that might make compliance about the rules better’ (female, 50, office worker, participant 88).  ‘I’d maybe feel a wee bit uncertain about the fact that this area had had a hotspot whilst everyone I can see when I’ve been out and about has been adhering to it. So, I would be a bit apprehensive after that’ (female, 51, teacher, participant 17). |
| Negotiations with friends, family members, or employers | Diagnosis, or suggestions to test from others | Personal convictions to test balanced against expectations and views of others | ‘I wasn’t going to get tested because I thought you needed two symptoms and I just had fever, but my wife said no even with one symptom you should go, so I said okay’ (male, 37, private sector manager, participant 104).  ‘My son had a cough which I was pretty sure was the aftermath of a cold that he’d had, and yes, I was going on whether what he had constituted a ‘continuous’ cough. But one of the teachers at school kind of commented and said, “Well, maybe you should get a test.” So, I had decided to order a test and keep him and my daughter off school (…) I chatted to my husband and yes, he kind of thought maybe it was a bit unnecessary, but I managed to persuade him that yes, it was the thing to do. So yes, and I guess my mum to some extent as well, I’d spoken to my mum about it’ (female, 52, public sector advisor, participant 101).  ‘I knew it was just a cold. I was 1000% sure it was negative but because nursery needs the test because of COVID, and so I had to do this […] it was a new-ish continuous cough. It was definitely a cold but I can’t prove it’s not COVID without a test’ (Female, 33, university lecturer, participant 51). |

### Table S4. Accessing tests

| **Challenges in accessing tests** | **Findings** | **Social and ethical considerations** | **Example quotes** |
| --- | --- | --- | --- |
| Booking system | Booking systems are sometimes experienced as arduous to navigate | Frustration with government over unacknowledged challenges of accessing tests | ‘I’d not tried to get [a test] posted before and I haven’t tried since, but it [the booking system] said it couldn’t verify my identity. I was really quite shocked by that because I would have thought that I am one of the most traceable, verifiable people that you could possibly have, and for me not to be identifiable was really quite alarming […] I tried it twice just to make sure I hadn’t entered anything in error, and both times it said that they couldn’t verify my identity [...] I gave up, to be honest, I didn’t try again’ (female, 32, student, participant 16).  ‘I think generally the information about testing has been very mixed, I don’t think there has been clarity and again this week, you’re always hearing about more and more messages of people trying to get tested and the system’s not working’ (male, 68, sports club staff, participant 81). |
| Transport logistics | Challenges getting to a testing centre  Available testing slots conflicting with, or undermining, other local or national guidance/restrictions. | Individual responsibility for not spreading infection through travel (e.g. public transport), or by crossing distances unnecessarily | ‘I got out the car to have a look at the whiteboard and realised that it was saying that that facility was closed and that you had to report somewhere else, which I wasn’t familiar with at all (…) There was no signage at all to help you get to where it was supposed to be. I was literally given a junction name off the motorway from this whiteboard and a sort of rough street name, and I managed to find it by sheer luck, actually. The signage was really bad’ (female, 38, teacher, participant 18).    ‘I ended up going down to Carlisle, driving 100 miles or whatever, having to stop for petrol, which was ridiculous, there were signs up saying, “Limited travel in North West England,” and they’d sent me there. I was really, really effed off with that, and thinking, “This is complete and utter chaos.” […] So, the testing, I think, is a shambles, or has been a shambles at times, or they created test centres and then not had them available, and I think the website’s failing. I think you shouldn’t be entering information so many times to find out it’s hundreds of miles away’ (female, 46, NHS staff participant 85).  ‘If you’ve driven to Galashiels with a five-year-old and they’ve had to wait an hour and a half for a test, it’s an awfully long time for that child not to go to the toilet, for example. Because that’s three and a half hours out of the house, and you shouldn't really be stopping on the way if you’ve gone for a COVID test’ (female, 42, private sector manager, participant 76).  ‘I was feeling quite dizzy and a bit nauseous so I didn’t really feel able to drive to the test centre. So, I tried to order one to get posted’ (female, 32, student, participant 16).  ‘At the time, we were both experiencing symptoms, we were alone in the north of Scotland. We had one car and the testing station was in Dundee and we were in Forfar. So being the responsible human beings we are, we were like, “No, we cannot do that because obviously we’ll just infect the people there” […] So we would have had to take the bus and obviously because it was flaring up, I was thinking, “Okay, we don’t want to do that, because that’s irresponsible’ (female, 19, student, participant 64).  ‘We’re just lucky that we have a car, we didn’t used to have a car. It would have been harder, like we would have had to just get the mail kit if we didn’t have a car” (Female, 33, university lecturer, Participant 51). |
| Working patterns | Testing often requires time off work | Obligations towards employers  Financial risks for the individual | ‘[My partner] and myself both had to take time off work to get tested and it ended up being almost half a day between the journey there and getting the testing and coming back and everything else, so it took a big chunk out of the day’ (male, 42, third sector manager, participant 72)  ‘I spoke to him [my line manager] the next morning and said, I messaged him early because I think I got the email about the results quite early, about eight in the morning or something like that. I was able to message him and say, “Look, the results have come back unclear, so I’m going to have to book another test and I’ve booked myself in this morning, so I’m going to be out of the office, I’m unavailable for an hour or so while I go and get that done.” And messaged a couple of people in my team just to make sure that they knew that I wasn’t going to be available for a little while, while I got the test’ (male, 37, private sector manager, participant 104).  ‘The ramifications of that false-positive test were awful last week. Our employer told us that we might not get paid. My whole family had to obviously go and get tested and take time off work’ (female, 53, private sector carer, participant 36). |

### Table S5. Sample collection

| **Factors** | **Findings** | **Social and ethical considerations** | **Example quotes** |
| --- | --- | --- | --- |
| Physical experience | Experiences of physical discomfort | Lack of acknowledgement of discomfort by government; government could do more to prepare people for testing/make testing easier | ‘It felt as if it was, for want of better phrase, tickling my brain; you know, rubbing into—the throat swab actually really made me sick. It was such a deep, deep throat swab that they took’ (female, 50, office worker, participant 88).  ‘[The drive-thru test] was really quite unpleasant, I was retching in the car and stuff. Again, up the nose, that was really sore as well, it goes right to the back, so kind of painful, uncomfortable, whereas at the GP I think it was only—I don’t know what, he did it differently, but it wasn’t sore or painful. It may be the fact he’s medically trained, but yes, that was much more pleasant as an experience’ (male, 28, public sector worker, participant 30).  ‘My son told me it is horrible, don’t do it unless you really have to. With the swab I’ve heard it’s like someone’s tickling your brain’ (male, 75, retired, participant 82). |
| Instructions and guidance | Concerns that one has not performed the test correctly | Duty to get oneself or one’s child tested, and individual responsibility for success of sample collection | ‘Because no one’s ever done it, so you don’t know what you’re doing at the beginning. You never—it actually took me half an hour to know what I’m doing. You have to read everything over and over (…) To be honest, it just makes you feel like a fool. You don’t know what you’re doing’ (male, 30, security guard, participant 42).  ‘So, none of the instructions she gave me were about the test, they were about what to do with it after. I think I’m fairly competent person with these things, but I can imagine other people being a bit more nervous, or a bit worried that they’re not doing it right’ (female, 36, university lecturer, participant 53). |
| Testing children | Parents feel unprepared to perform what is felt to be a difficult and potentially distressing medical procedure. | Parental responsibility for children’s physical and emotional wellbeing  Frustration with government over hidden challenges of testing | ‘I knew a lot about it, but it was a lot more traumatic to do it on a child and it was traumatic for my children […] When I saw the instructions, it was just a mismatch between what you have to do as a parent and the way that it was being talked about in the instructions. There was a drawing where the instructions made you feel like you should be able to do this happy thing and make a funny face and give a toy or do something and it’s going to work. And I did all of those things with my first son and as soon as I started the test, he just freaked out’ (female, 33, university lecturer, participant 51).  ‘I’ve had friends who have autistic children who have been really distraught by the testing […] They’re not going into the system because it was so stressful and upsetting for them’ (female, 46, NHS staff, participant 85).  ‘Then my son was much more difficult. He was nervous anyway. He gets a bit nervous about any kind of tests or vaccination or whatever. It was really difficult, he just kept gagging every time […] Luckily, I’d just done 10 seconds and then he vomited all over himself. He was crying and I was like, “That’s nice, but now I need to stick it up your nose.” Luckily it wasn’t a huge amount, but he had some vomit down his front. Luckily it didn’t go on the swab, otherwise we would have had to start again. And then his nose was similar. He was saying it was sore, but I think he was just by that point a bit stressed and upset’ (Female, 36, university lecturer, participant 53).  ‘He wasn’t happy at all having it done, but we knew that it would be unpleasant, so we brought lots of snacks and distractions and he was fine within a minute, whereas we still felt fairly traumatised for having to hold him while the other one did it’ (female, 41, NHS staff, participant 96).  ‘I suppose I was more concerned that I did something wrong that invalidated the test and then I really, really didn’t want to have to go and do it again’ (female, 38, university teacher, participant 18).  ‘The other friend also…. Her and the friend that’s got an autistic child who was really scared by obviously shoving things up her nose. They’re all just like, “well, if it comes up again, we’ll just self-isolate, two weeks and we’re not going to do anything”. So, they’ve been cut off. They’re not going into the system because it was so stressful and upsetting for them. That’s difficult, because then their information obviously is lost to Test and Protect or whatever, so people are just not going to be open about it, I guess.’ (female, 46, NHS staff, participant 85). |
| Absence of health professionals | Concern about accuracy of self-tests and reliability of population level testing data and policy making | Distrust of testing method linked to distrust of government response.  Duty of government to provide testing expertise and accessibility | ‘I think if they were being administered by a nurse then, particularly for children, I’d have a bit more confidence that they were going to be accurate […] But for children, where it’s being administered by a parent, it’s kind of a tough spot to be in—to administer a very uncomfortable, painful test to your own child. So, to then expect parents who aren’t trained to do it accurately … leaving it to the parents makes me think that I really don’t believe that they’re accurate’ (female, 38, business owner, participant 75).  ‘It probably helps if it’s a health professional involved in that, it might give somebody some confidence that people know what they’re doing and it’s not just Joe Bloggs poking you with a stick. It might help from some public confidence point of view’ (female, 39, NHS staff, participant 09).  ‘I just think I would worry about the reliability of self-swapping, to be honest […] I don’t think the self-testing is reliable and the data, you know, I certainly wouldn’t take it at its value, to be honest […] If I hadn’t had that test done, there’s no way I would be able to put that up my nose and swab my throat as deeply as they did for me’ (female, 50, office worker, participant 88).  ‘When the testing team came to the flat the nurse did the swab and it was very gentle. When the test came back negative we were so confused because we were sure we must have it. Later I had a swab at a drive thru centre and it was so uncomfortable and invasive it made me more concerned that my first test had not been done properly and probably wasn’t accurate’ (female, 32, student, participant 16).  ‘I think the statistics are out there, I think the fact they changed the methodology of how they report them and I’ve noticed they’ve changed it again in the last week […] it makes you apprehensive in believing it all. I think if the information is not collected properly and some of it is lost in translation. I think the emphasis keeps changing, the parameters they’re changing to maybe make things sound better or worse, according to what agenda they want to run’ (male, 68, sports club staff, participant 81). |

### Table S6. Waiting for, receiving, interpreting and acting on results

| **Challenges** | **Findings** | **Social and ethical considerations** | **Example quotes** |
| --- | --- | --- | --- |
| Self-isolating while awaiting results | Ambivalence about self-isolating while awaiting a test result. | Lack of recognition by government of personal costs of testing/isolating  Ethical obligation to isolate balanced with personal circumstances and/or social obligations to others. | ‘Waiting for a test result meant we couldn’t celebrate my mother’s birthday’ (female, 56, public sector worker, participant 99)  ‘I think obviously going into self-isolation immediately after a test and stuff, if I wasn’t prepared for it, it might cause issues, just in terms of making sure I had enough food and stuff. But as well as that obviously I wouldn’t be at work and we’re a bit short-staffed as it is, and I would be on – I think sick pay is still only £94 a week, which isn’t great. It’s not a lot of money’ (female, 38, teacher, participant 18).  ‘I have to confess my husband went to work. My husband’s work actually, they have offered, if you don’t take any time off due to self-isolation, COVID, et cetera, they are giving people a two-week wages bonus round about Christmas time, which is – I think now that that’s pretty bad, because as a result of that, as he didn’t want to miss work. So yes, he went to work’ (female, 52, public sector worker, participant 101).  ‘I don’t think we completely isolated, I think there were a few times where we went shopping, which isn’t technically correct, but just because he was so convinced and he was a little bit annoyed about the whole situation. So he was like, “No, I’m going out.” Other than that, we were quite strict and obviously at the time as well, we didn’t know how long it was going to take’ (female, 19, student, participant 64).  ‘For instance, instead of being able to take the dog out for a proper walk, he just had to go to the garden, and he had to do his motions out there’ (male, 42, third sector manager, participant 72).  ‘I mean, if we’d had just had my son tested, once it came back, we’d have carried on. Certainly, I know my sister has had her wee boy tested and they just went on his result, in terms of stopping the self-isolation’ (female, 41, NHS staff, participant 96). |
| Negative test results | Negative test results are experienced as reassuring, and enable a return to work, and social obligations to be fulfilled | Responsibility to protect friends, colleagues, and wider community | ‘I suffer from anxiety, which I didn’t actually before the pandemic. I suppose on a personal level, having a test and having a negative result reassured me […] It definitely gave a sense of reassurance and kind of relief that we weren’t spreading it either’ (male, 28, public sector worker, participant 30).  ‘I’d like to know every carer, whether it’s one who is in a nursing home or one who visits people in their own home, I would like to know they are being tested regularly’ (female, 85, retired, participant 25).  ‘I think after I had the result, I definitely way more like at peace with the fact that I could just socialise. Because as well my nieces and nephews and my grandmother as well, just in general it just made me more calm that I wasn’t a potential vector for all of these family members and stuff. So, in that sense I think it did change my behaviour’ (male, 30, student, participant 68).  ‘Emotionally it was a relief because the children didn’t have it and it meant that we were therefore not likely to get it. Because obviously you live in the same house. By the time they’d got it, it’s a little bit difficult to isolate from them in the same house […] emotionally for the kids it was brilliant. They could go straight back to school, which was what they wanted’ (male, 50, participant 92) |
| Trust in results | People can feel doubt in testing when test results do not align with their diagnostic suspicions | Responsibility for self-diagnosis | ‘They [test results] both came back negative, which was really very puzzling to us. It’s still very puzzling to me, like how we came into direct contact with somebody who stayed at the flat, and not to get it seemed really quite wild’ (female, 32, student, participant 16).  ‘A little bit of me was worried because it was self-administered […] you know, the level of false negatives of the self-administered test, so that’s what I was trying to avoid […] I’d seen how badly [my daughter] was gagging, trying to find the back tonsils and then trying to do the nasal bit. So, it’s a possibility she didn’t do it properly. I was 85 per cent sure that was correct’ (female, 46, NHS staff, participant 85).  ‘When I got the third test, it was a male nurse and I think he was ex-Army, and he basically got me in a headlock and shoved the thing right down my throat. I really felt as though I’d been tested after he’d done it, he wasn’t gentle but he got the job done and he said, “Right, given that your last two tests have come back negative, this test is going to Edinburgh Royal Infirmary rather than being at [the hospital’s] own lab’ (female, 63, public sector worker, participant 93).  ‘He didn’t have a cough or anything like that and I know a wee bit about what the rates are at the moment and that there’s fairly low chances. And then, I think my dad told me that it was about one in a hundred tests coming back positive, so that reassured me further that it probably wasn’t’ (female, 41, NHS staff, participant 96). |
| Self-isolating after a positive test in an immediate contact | Strong desires to self-isolate if instructed to do so, but imagined challenges  Uncertainty over whether to follow guidelines | Ethical obligation to isolate to prevent transmission and protect others in the community, balanced with personal circumstances, and other duties of care to family members, employers and the general public. | ‘It is on my mind quite a lot, that there is a potential that at some point, the test and trace are going to call me to say, “As a household you need to isolate and if you develop symptoms, get a test”. Now my concern is obviously about I really don’t want that for my girls, because they’ve lost so much school already this year, I don’t want them to lose any more school. I think they are falling so behind.  So that would really – it would make me anxious, you know, having to self-isolate. But, at the same time, you have a responsibility for the health and well-being of everybody. So, I absolutely would do that’ (female, 50, officer worker, participant 88).  ‘I could imagine feeling pretty conflicted about whether to actually stay home for two weeks [if called by Test and Trace] because what are really the chances you got it from one person who was in the pub that time who later tested positive? I think it would be quite easy to rationalise not doing the full two weeks, especially if you then felt fine’ (female, 30, student, participant 57).  ‘So I wasn’t very good at self-isolating because I had to go out a couple of times. One time was a biker, a mountain biker, who fell over and got injured and needed emergency help and I was close by so I went out, because I live in the [area], so I went out and helped him, even though I was supposed to be isolating. So, my one attempt at isolating wasn’t very good’ (male, 45, outdoor public sector worker, participant 50).  ‘I think probably if you’re quite new then you wouldn’t probably get paid. But if you’ve been there for a while, then you probably will get paid. But I can’t see the company paying us if we were off. I think that’s another reason people are scared to get tested, in case they were positive. Actually, if people were positive, people would maybe still come to work, because people have got expenses to pay and if companies are not – we’ve not been offered anything, because we’ve not been in that position to know’ (male, 30, security guard, participant 42). |
